# Food insecurity amongst Canadian children with food allergy during the COVID-19 pandemic

**DOI:** 10.1186/s13223-025-00958-3

**Published:** 2025-03-30

**Authors:** Zoe Harbottle, Jakob Pettersson, Michael A. Golding, Marina Jonsson, Leslie E. Roos, Jennifer L. P. Protudjer

**Affiliations:** 1 Department of Pediatrics and Child Health, Rady Faculty of Health Sciences, Max Rady College of Medicine, University of Manitoba, Winnipeg, MB Canada; 2 Children’s Hospital Research Institute of Manitoba, 501G-715 McDermot Ave, Winnipeg, MB R3E 3P4 Canada; 3 Department of Food and Human Nutritional Sciences, Faculty of Agricultural and Food Sciences, University of Manitoba, Winnipeg, MB Canada; 4 Karolinska Institutet, Stockholm, Sweden; 5 Institute of Environmental Medicine, Karolinska Institutet, Stockholm, Sweden; 6 Centre for Occupational and Environmental Medicine, Stockholm County Council, Stockholm, Sweden; 7 Department of Clinical Science and Education, Karolinska Institutet, Södersjukhuset, Stockholm, Sweden; 8 Department of Psychology, Faculty of Arts, University of Manitoba, Winnipeg, MB Canada

**Keywords:** Food allergy, Food insecurity, Pediatrics

## Abstract

Food insecurity is a growing concern, that is currently estimated to affect 1 in 4 Canadian children. Due to the additional effort required for management and the disproportionate cost of allergy friendly foods, households with food allergy may be at increased risk of experiencing food insecurity. With this in mind, we aimed to describe and compare the prevalence of food insecurity amongst children in households managing pediatric food allergy between 2019, 2020 and 2022 using a repeated cross-sectional design. A total of 117 participants were recruited via social media between these three distinct timepoints, referred to as waves. All participants completed an anonymous online survey consisting of demographic questions and the Household Food Security Module from the Canadian Community Health Survey. Rates of child food insecurity were comparable between Waves 1 and 2 (34% and 35%, respectively; p=0.75), but, increased significantly between Waves 2 and 3 (35% and 56%, respectively; p=0.005). Amongst children identified as food insecure, the proportion who were marginally food insecure remained relatively stable, whereas, levels of moderate food insecurity appeared to increase, although not significantly. Conversely, the proportion classified as severely food insecure decreased across the waves, but again, this difference was not statistically significant. Our findings demonstrate an upward trend in child food insecurity levels, showcasing the need for a larger scale, longitudinal evaluation of the intersection between food allergy and food insecurity. We call on researchers and policy makers to attend to this important issue.

To the Editor:

Food insecurity, defined by the Government of Canada as “the inability to acquire or consume an adequate diet quality or sufficient quantity of food in socially acceptable ways, or the uncertainty that one will be able to do so” [1], is a widespread issue in Canada with 1.8 million children, or 1 in 4, living in a food-insecure household [2]. Childhood food insecurity can negatively impact mental health, physical health, and educational attainment [3, 4].

Households managing food allergy face considerable burdens due to the additional effort required for management and the disproportionate costs of allergy-friendly products. In the year prior to the COVID-19 pandemic, households with food allergy reported spending approximately $200 Canadian more per month on food compared to households without food allergy [5]. These additional costs further increased during the early months of the pandemic with food-allergic households reportedly spending an additional $100-$200 per month on food than in the months prior to the pandemic [6].

Considering the significant financial burden of food allergy, it is reasonable to speculate that families with food allergy may be at a greater risk of being food insecure than families without food allergy. Indeed, we previously reported that the prevalence of food insecurity amongst adults with a child with food allergy living in their home increased between 2019 (i.e. the year prior to the COVID-19 pandemic) and 2022, from 22.9 to 74.4% [7].

In the present study, we aimed to describe and compare the prevalence of food insecurity amongst children in households managing food allergy between 2019, 2020 and 2022.

To evaluate the relationship between food allergy and childhood food insecurity in the year prior to the pandemic, 2019, through to 2022, we employed a repeated, cross-sectional design. Using this design, data was collected from three separate samples at three time points, hereafter referred to as waves, via online anonymous survey. All participants had at least one child with food allergy, and were recruited via social media, including X (formerly Twitter), Instagram, and Facebook. Wave 1 data was collected between April and May 2020 and was based on participants’ experiences in 2019. Wave 2 data was collected between March 2020 and December 2020 and was based on participants’ experiences at the beginning of the COVID-19 pandemic. Wave 3 data was collected between January and May 2022 based on participants’ experiences in January 2022. At all three waves, data collected included demographic information and data on food security, measured using the Canadian Community Health Survey Household Food Security Survey Module (CCHS HFSSM). Of the CCHS HFSSM’s 18 questions, 8 evaluate child food security and are the focus of the present study (see Table 1 for questions). Children were categorized as food secure if they reported zero affirmative answers, and food insecure if they reported one or more affirmative answers [8]. Those identified as food insecure were then placed into three subcategories; marginally food insecure (1 affirmative answer), moderately food insecure (2 to 4 affirmative answers) and severely food insecure (5 to 8 affirmative answers) [8]. Differences in both the proportion of participants classified as food insecure and the proportion falling in each of the three food insecurity categories (marginal, moderate, and severe) were compared across the three time-points using Chi-squared tests, unless the cell counts were below 5 in which case the Fisher exact test was performed. A p-value of < 0.05 was considered statistically significant. Data analysis was conducted using Stata (Version 18, College Station, TX).

**Table 1:** Questions from the CCHS household food security survey module pertaining to child food insecurity

You and other adults in your household relied on only a few kinds of low-cost food to feed the child(ren) because you were running out of money to buy food. Was that often true, sometimes true, or never true since the start of the COVID-19 pandemic?	a. Often true b. Sometimes true c. Never true
You or other adults in your household couldn’t feed the child(ren) a balanced meal, because you couldn’t afford it. Was that often true, sometimes true, or never true since the start of the COVID-19 pandemic?	a. Often true b. Sometimes true c. Never true
Child(ren) were not eating enough because you and other adult members of the household just couldn’t afford enough food. Was that often true, sometimes true, or never true since the start of the COVID-19 pandemic?	a. Often true b. Sometimes true c. Never true
In the past 12 months, did you or other adults in your household ever cut the size of any of the children’s meals because there wasn’t enough money for food?	a. Yes b. No c. Don’t know / refuse to answer
Since the start of the COVID-19 pandemic, did any of the children ever skip meals because there wasn’t enough money for food?	a. Yes b. No c. Don’t know / refuse to answer
How often did this happen?	a. Yes b. No c. Don’t know / refuse to answer
Since the start of the COVID-19 pandemic, were any of the children ever hungry but you just couldn’t afford more food?	a. Yes b. No c. Don’t know / refuse to answer
Since the start of the COVID-19 pandemic, did any of the children ever not eat for a whole day because there wasn’t enough money for food?	a. Yes b. No c. Don’t know / refuse to answer

Abbreviations: CCHS: Canadian community health survey; COVID-19: coronavirus disease 2019.

Data collection was approved by the University of Manitoba (Wave 1: Research Ethics Board (HS23849 P2020:030); Waves 2 and 3: Health Research Ethics Board (HS24604 H2021:034)).

A total of 41, 49, and 27 households were included in Waves 1, 2, and 3, respectively. All households reported having at least one child with food allergy within the household (Table 2). Results revealed comparable rates of child food insecurity between Waves 1 and 2 with 34% and 35%, respectively (*p* = 0.75). However, food insecurity significantly increased to 56% between Waves 2 and 3 (*p* = 0.005) (See Fig. 1).

**Table 2:** Participant characteristics for each of the three waves

		Wave 1 (*N* = 41)					Wave 2 (*N* = 29)							Wave 3 (*N* = 27)								
		n		%			n	%		M ± SD				n		%		M ± SD				
**Number of children in household**																						
*1*		15		37.5			16	33.3						10		37.0						
*2*		16		40			25	52.1						14		51.8						
*3 or more*		9		22.5			7	14.6						3		11.1						
*Mean child age ± standard deviation*		–		–			47	-		5.6 ± 4.7				27		-		7.4 ± 4.7				
Aged 0 to 18 months		17		41.5			–	–						–		–						
Aged 18 months to 4 years		21		51.2			–	–						–		–						
Aged 5 to 8 years		18		43.9			–	–						–		–						
Type of food allergy *																						
*Milk*		13		31.7			23	47.9						11		40.7						
*Eggs*		9		22.0			14	29.2						6		22.2						
*Peanuts*		6		14.4			23	36.7						12		44.4						
*Tree nuts*		3		7.32			18	36.7						6		22.2						
*Fish*		2		4.9			6	12.5						2		7.4						
*Shellfish*		1		2.4			4	8.3						3		11.1						
*Soy*		1		2.4			4	8.3						3		11.1						
*Wheat and/or triticale*		1		2.4			3	6.2						1		3.7						
*Sesame*		0		0			–	–						–		–						
*Mustard*		0		0			1	2.1						0		0						
*Other*		13		31.7			9	18.8						4								
**EAI possession**		17		41.5			33	76.7						11		44						
**Highest level of education among caregivers**																						
*High school*		5		12.2			5	12.0						5		19.2						
*College*,* Trade school or Undergraduate*		27		65.8			21	50.0						8		30.8						
*Graduate or Professional degree*		9		22.0			16	38.1						13		50.0						
**Annual household income 2019 (CAD)**																						
*<30*,*000*		7		18.9																		
*30*,*001–60*,*000*		6		16.2																		
*60*,*001–90*,*000*		9		24.3																		
*90*,*001-120*,*000*		5		13.5																		
*>120*,*000*		10		27.0																		
**Annual income in CAD during COVID-19**																						
*< 65 000*							11	26.2							8		30.8					
*65 001–100 000*							10	23.8							4		15.4					
*100 001–200 000*							16	38.1							10		38.5					
*> 200 000*							5	11.9							4		15.4					

EAI = Epinephrine auto injector CAD = Canadian Dollars M = Mean N = Number SD = Standard Deviation

* Not mutually exclusive

**Fig. 1:**
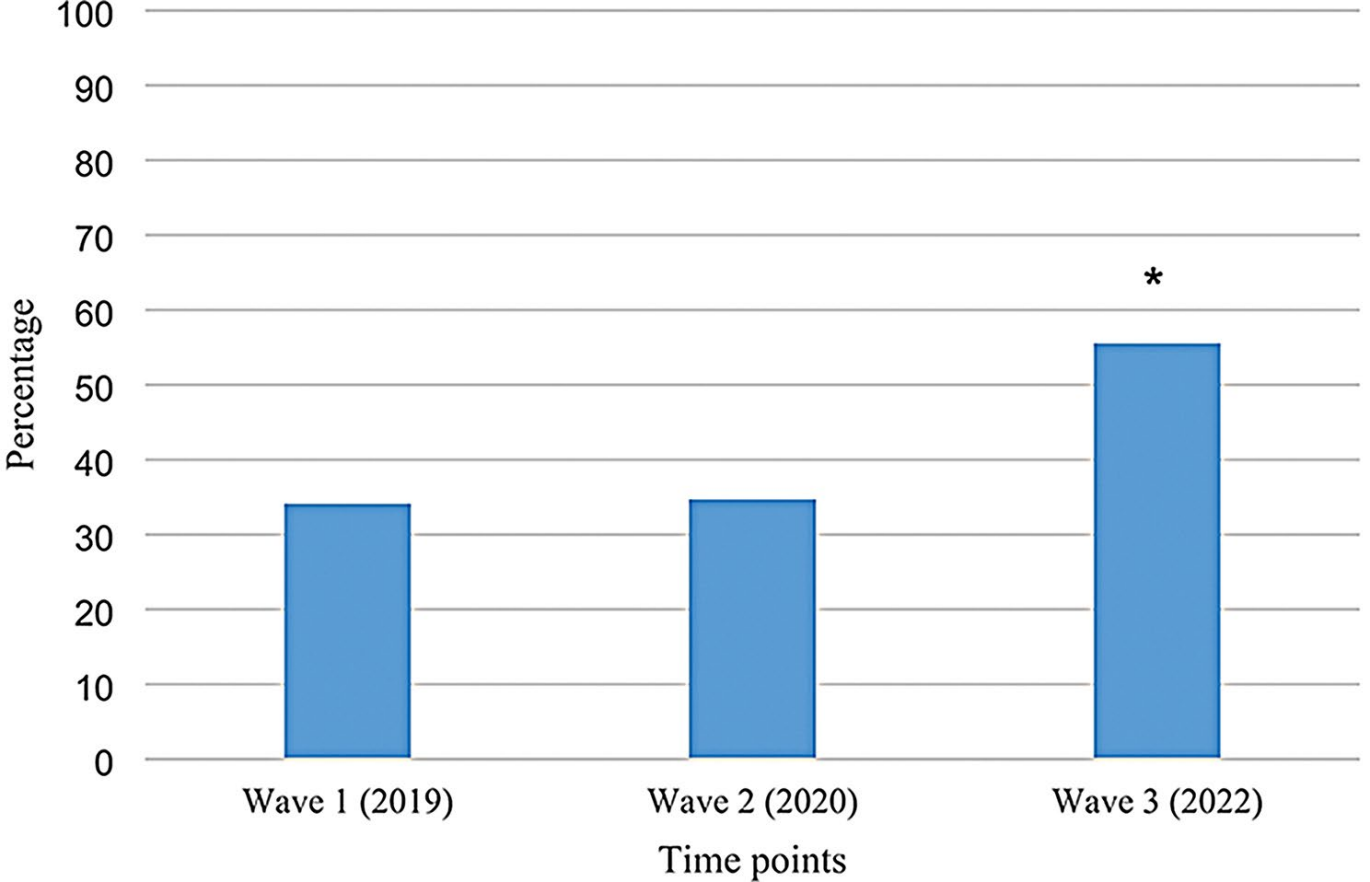
Prevalence of food insecurity amongst Canadian children with food allergy between 2019 and 2022. p=0.75 between 2019 and 2020; p=0.005 between 2020 and 2022. *p=0.005 vs. Wave 2

Amongst children identified as food insecure, the proportion who were marginally food insecure at each of the three waves remained relatively stable (21%, 18% and 20% in Waves 1, 2 and 3, respectively; *p* = 0.90). In contrast, moderate food insecurity appeared to increase, but this increase was not statistically significant (36%, 47% and 67% in Waves 1, 2 and 3, respectively; *p* = 0.52). Conversely, severe food insecurity appeared to decrease across the waves (43%, 35%, and 13% in Wave 1, 2 and 3, respectively; *p* = 0.51), but again, this change was not statistically significant (See Fig. 2).

**Fig. 2:**
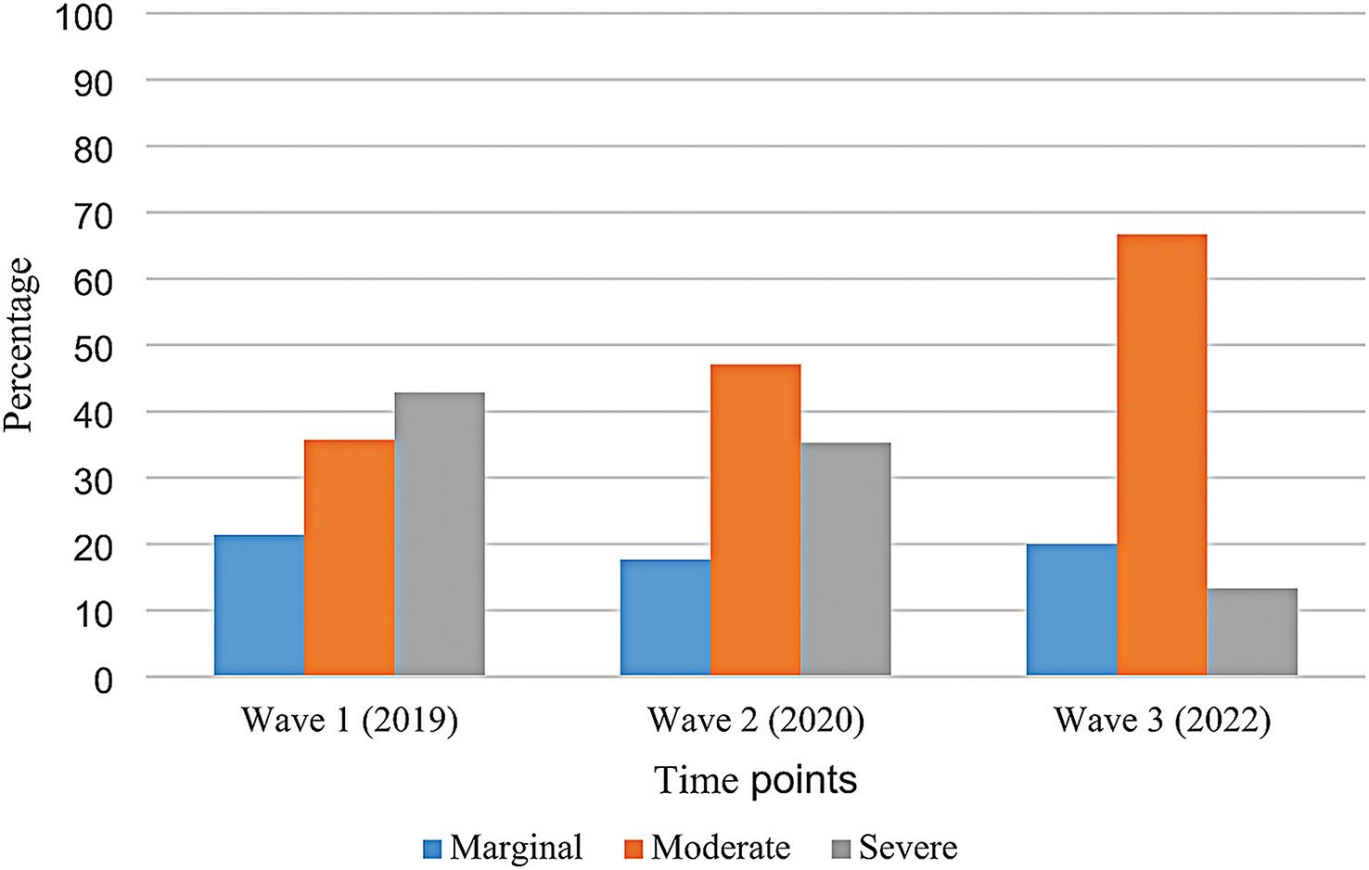
Prevalence of marginal, moderate, and severe food insecurity amongst Canadian children with food allergy and food insecurity between 2019 and 2022

This is the first study to evaluate childhood food insecurity in Canadian households managing pediatric food allergy that provides a comparison between prior to and during the COVID-19 pandemic. While our findings did not demonstrate a significant change in rates of food insecurity between the initial months of the pandemic and the year prior, we did find a significant increase in rates of food insecurity between 2020 and 2022 among Canadian children with food allergy. During the same time frame, Canadians faced large increases in grocery prices and the loss of emergency pandemic relief benefits, both of which may have contributed to changes in food security status.

We acknowledge the limitations of our study. Most notably, this study did not use a true repeated measures design. Therefore, it is possible that the differences in food insecurity rates between waves stem from differences in the samples rather than changes in food security status over time. Additionally, all recruitment occurred via social media, limiting participation to individuals with internet access. This may cause an underrepresentation of households suffering from more severe levels of food insecurity and an overrepresentation of wealthier households. Finally, the sample size for each of the waves was relatively small, increasing the potential that food security levels may not generalize to the larger population.

In conclusion, rates of co-existing food insecurity and food allergy increased between 2020 and 2022, but no significant differences were found across waves within the various subcategories of food insecurity. With food prices beginning to stabilize in the wake of the COVID-19 pandemic, more large-scale, longitudinal research is needed to better understand the relationship between changes in food price and availability and food security among families managing food allergy. This information will better equip policy makers and non-profits to provide food-allergic families struggling with food security with the help they need.

## Data Availability

Requests for aggregate data may be submitted to the corresponding author. Requests will be discussed by a minimum of two authors.

## Abbreviations

CCHSHFSSM: Canadian Community Health Survey Household Food Security Survey Module
